# Performing a sperm DNA fragmentation test in addition to semen examination based on the WHO criteria can be a more accurate diagnosis of IVF outcomes

**DOI:** 10.1186/s12894-023-01257-y

**Published:** 2023-04-29

**Authors:** Tsuyoshi Okubo, Noriyuki Onda, Teruaki Hayashi, Tatsuya Kobayashi, Kenji Omi, Tomoya Segawa

**Affiliations:** 1 Shimbashi Yume Clinic, 2-5-1 Shimbashi Minato-ku, Tokyo, 105-0004 Japan; 2 Department of Reproductive Medicine, Graduate School of Medicine, Chiba University, 1-8-1 Inohana Chuo-ku, Chiba, 260-0856 Japan

**Keywords:** Human spermatozoa, Semen examination, Sperm DNA fragmentation, Sperm morphology, Infertility of male, Blastocyst development

## Abstract

**Background:**

We analyzed the sperm DNA fragmentation index (DFI) and general semen test based on the World Health Organization (WHO) criteria and compared the two tests using semen factors. In addition, we examined whether DFI is a reliable parameter associated with in vitro fertilization (IVF) outcomes.

**Methods:**

Sperm chromatin dispersion (SCD) and general semen tests were conducted in accordance with the WHO 2010 guidelines, and correlations between the two tests were investigated. The WHO criteria were set as the cutoff values for each of the following factors: semen volume, concentration, total sperm count, motility, and normal morphology, and compared with the DFI results.

**Results:**

The subjects had a mean sperm DFI of 15.3% ± 12.6%, and the DFI increased with age. In contrast, motility and normal morphology decreased as the DFI increased. Patients who satisfied the WHO criteria in terms of concentration, total sperm count, and motility had a significantly lower DFI than those who did not satisfy the criteria. Therefore, evaluation with a general semen test based on the WHO criteria should be regarded as a qualitative evaluation of all factors other than semen volume and normal morphology.

**Conclusions:**

High DFI (≥ 30%) caused a low blastocyst development rate following intracytoplasmic sperm injection. Male infertility due to DFI should be suspected when IVF results are poor despite normal semen findings based on the WHO criteria. The results of this study suggest that the SCD test may more accurately evaluate the correlation between IVF clinical outcomes and male infertility. Therefore, it is important to focus on DFI measurements.

## Background

Female factors are important in determining the success of in vitro fertilization (IVF). However, many reports have shown that male factors also affect the success or failure of IVF, indicating that male examination is important for assessing fertility in couples [1, 2]. Generally, male fertility is assessed using semen analysis; semen tests are based on diagnostic methods in line with the WHO criteria and include analysis of semen volume, sperm concentration, total sperm count, sperm motility, and sperm malformation rate [3, 4]. It is well known that conventional sperm motility analysis is dependent on the subjectivity of the observer, and the diagnostic sensitivity of semen motility analysis for assessing fertility potential is low. Hence, only motility of sperm cannot represent the fertility of sperm. In addition, evaluation of the malformation rate is also subjective, as there could be individual differences, and is based only on an approximate morphological sperm evaluation viewed through a biological microscope. Therefore, sperm evaluation criteria based on conventional semen analysis methods alone may not be a primary factor in determining subsequent IVF embryonic potential and pregnancy and miscarriage rates after transplantation. In addition to evaluating the number and motility of sperm, factors that contribute to infertility, such as whether sperm has the ability to fertilize oocytes and how they are involved in embryogenesis after fertilization, should be evaluated.

In our hospital, we automatically evaluated concentration and motility using a sperm analyzer system based on the WHO criteria and sperm morphology via the Kruger test [5]. The methods or recommendations for semen analysis are limited to monitoring sperm count, motility, and morphology. Further and deeper testing is needed to improve our understanding of the etiology of male infertility. Abnormal alterations occurring during sperm chromatin configuration or histone-to-protamine exchange can lead to sperm DNA fragmentation (SDF) [6]. The SDF has been shown to be associated with fertilization failures, delayed embryo development, and implantation failures. Therefore, we considered the DFI by SCD test [7–9]. DFI is based on sperm chromatin nuclear analysis [10]. The aim of DFI was to evaluate the qualitative factors of sperm nuclei. Methods for detecting DFI include the TUNEL assay (fluorescence microscopy with terminal deoxynucleotidyl transferase-mediated deoxyuridine triphosphate-nick end labeling), comet assay (detection with single-cell gel electrophoresis), AO test (fluorescence microscopy using acridine orange, a nucleic acid fluorescent dye), and SCSD test (sperm chromatin structure assay). However, DFI analysis techniques require sophisticated equipment and are expensive. In this study, DFI analysis was performed using the SCD test based on chromatin structural analysis of the sperm nucleus [11], and quantified using the Halo sperm DNA kit [12]. This kit is commercially available and enables relatively simple and low-cost DFI analysis. In addition, the SCD test method is equivalent to or more sensitive than the TUNEL method for the analysis of fragmented sperm DNA. The protamine in sperm nuclei with less DNA fragmentation forms a halo in the DNA strands extracted using acid and detergent treatment. The principle of measurement utilizes the fact that halo formation is inhibited in fragmented sperm DNA. DFI is calculated as the number of non-halo-forming sperm in the total number of sperms and is expressed as a percentage [13].

In this study, we investigated the correlation between DFI tests and semen analyses based on WHO criteria using the following factors: semen volume, concentration, total sperm count, motility, and normal morphology. In addition, we compared each factor by separating the subjects into two groups: the group with measurements below the WHO criteria and the group with measurements at or above the WHO criteria. The WHO criteria were set as the cutoff values for each of the following factors: semen volume (≥ 4 mL), concentration (≥ 15 × 10^6^ mL), total sperm count (≥ 39 × 10^6^), motility (≥ 40%), and normal morphology (≥ 4%). We investigated the clinical significance of the SCD test in the strict assessment of male infertility during IVF.

## Methods

### Study population

This retrospective cohort study included patients who visited the Shimbashi Yume Clinic between June 2020 and June 2021. A total of 182 male patients with the main complaint of infertility who requested semen testing were included. All female patients whose male partners had an SCD test underwent IVF treatment by ICSI. Written informed consent was obtained from all the patients. This study was approved by the ethics review board of our hospital (SYC2021-8). The tests included in the study were automatic sperm motility analysis using SMAS (DETECT, Japan), Kruger test via the Diff-Quik method, and DFI test using the SCD method.

### Semen examination and analysis

#### Sperm motility test (general semen test)

Semen volume was measured after it was sufficiently liquefied, and the concentration and motility were measured using SMAS.

#### Sperm morphology (Kruger sperm function test)

Sperm were treated using the Diff-Quik Staining Kit (Sysmex Corporation, Japan). Normal morphology was analyzed using 200 or more sperms under a biological microscope (× 400), and the head, midpiece, and tail of the sperm were examined in detail.

#### SDF test

The SCD test is also known as the halo assay. The proportion of sperm with halos to the total number of sperm was calculated as the DFI, and was evaluated using a Halosperm DNA kit (HT-HS10, Halotech DNA, Spain). DNA fragmentation in sperm nuclei can be quantified using this kit. In normal sperm, halos formed by the loop strands of DNA in the head are visible, but halos do not form in the loop strands of damaged DNA of fragmented sperm. The ratio of fragmented sperm to the total number of sperm was analyzed and expressed as the DFI ratio using a selection of 300 or more sperms. A DFI value of 30% was used to differentiate between fertile and infertile human sperms.

#### IVF outcome

In total, 2803 oocytes were retrieved from 182 female patients. Oocyte maturation status was defined by the first polar body visualization or meiotic spindle confirmation. Oocytes were inseminated using ICSI sperm injection. DFI was assessed in the following groups: low (≤ 15%), medium (15–30%), and high (≥ 30%). The outcomes were normal fertilization, multiple nucleation (3PN <), and good-quality blastocyst formation rates (Gardner criteria: grade > 3).

### Statistical analysis

For patient characteristics, summary statistics were constructed using frequencies and proportions for categorical data and means, standard deviations (SDs), and ranges for continuous variables. Pearson’s correlation test was used to determine the correlation between parameters. Data from each group are expressed as the mean ± SD, and the mean values of each group were compared using ANOVA. The rates of 2PN and good blastocyst formation were compared using the χ^2^ test. We performed multivariate logistic regression analysis using confounding variables. We chose the confounding factor to minimize Akaike's Information Criterion. We used DFI (high, medium, or low), sperm normal morphology rate, women’s age, and oocyte stage (MII, MI, or GV) at oocyte retrieval to analyze ICSI outcomes, and women’s age, DFI (high, medium, or low), blastocyst vitrification time, ICM, and TE grade to analyze embryo transfer outcome as a confounding factor. Statistical significance was set at p < 0.05. Statistical analyses were performed using the JMP Pro 15 software (SAS Institute Inc., USA).

## Results

The mean age of the 182 male patients at the time of the sperm tests was 40.9 ± 5.7 years (range, 22–53 years). All the patients were examined at our hospital for the first time. Semen volume and concentration, total sperm count, and motility were measured using a general semen test. Normal morphology was examined using Kruger test. DFI ratios were determined using the SCD test (Table 1).

**Table 1:** General semen findings and DFI in target patients

Factors	Mean ± SD	Range
Age (years)	40.9 ± 5.7	22–53
Semen volume (mL)	2.9 ± 1.3	0.3–8.3
Concentration (10^6^/mL)	78.1 ± 66.8	0.5–410.0
Total sperm counts	222.2 ± 203.2	1.3–1054.2
Motility (%)	58.1 ± 19.7	0–93.2
Normal morphology (%)	2.8 ± 1.7	0–10.0
DFI (%)	15.3 ± 12.6	1.2–97.0

*Data presented as mean ± standard deviation

**Fig. 1:**
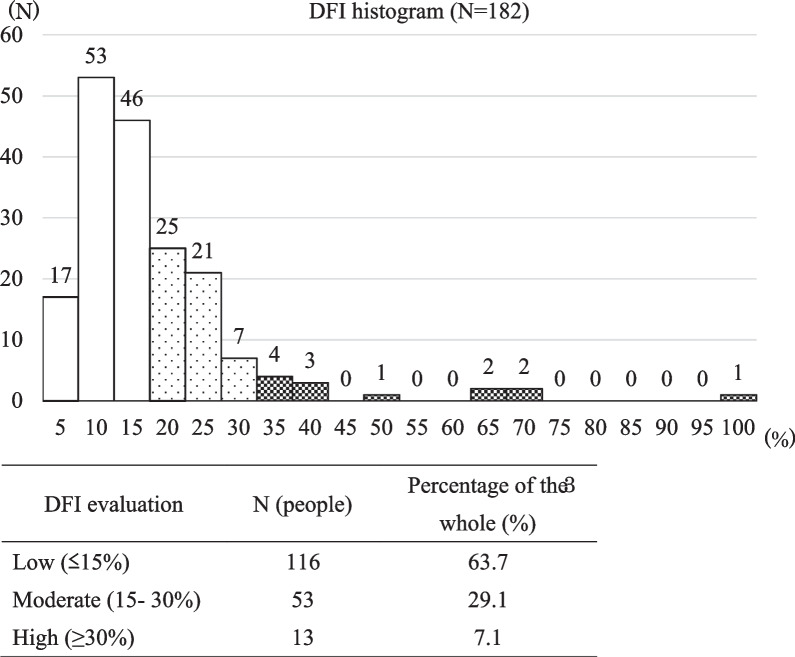
DFI distribution and percentage for each evaluation. The mean DFI was 15.3% ± 12.6%, with a normal DFI rating of 63.7%. On the other hand, samples with moderate and high DFI evaluations accounted for approximately one-third or more. The highest DFI was 97.0% (range, 1.2–97.0%)

The mean DFI was 15.3% ± 12.6%. Using the Halo sperm HT-HS10 DNA kit per the instructions provided in the package insert, 7.1% (13/182) of the patients had a high DFI (≥ 30%) (Fig. 1).

The DFI tended to increase with age (r = 0.2995, p < 0.0001), while motility and normal morphology tended to decrease as the DFI increased (Table 2).

**Table 2:** Various factors in general semen tests and correlation with DFI

Factor	DFI	
	Correlation (r)	p value
Age (years)	0.2995	< 0.0001
Semen volume (mL)	0.0450	0.5463
Concentration (10^6^/mL)	− 0.121	0.1037
Total sperm counts	− 0.0639	0.3913
Sperm motility (%)	− 0.5405	< 0.0001
Normal morphology (%)	− 0.2582	< 0.001

*p < 0.05 is considered as significant by Pearson correlation co-efficient test

**Fig. 2:**
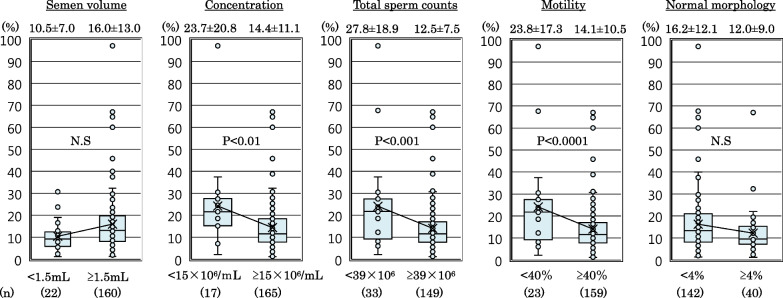
DFI comparison in the group at or above and the group below the WHO criteria for each factor in the general semen test. There was no significant difference in DFI for semen volume between the groups at or above the criteria and those below the criteria. Regarding concentration, motility, and normal morphology, the group below the criteria (that is, the group that did not meet the WHO criteria) had a higher DFI

A comparative examination of DFI against the WHO criteria did not reveal a significant difference between the group at or above the criteria and the group below the criteria for semen volume (p = 0.0515) and normal morphology (p = 0.0693). However, for concentration, total sperm count, and motility, those below the criteria (that is, those that did not meet the WHO criteria) tended to have a higher DFI than that the group at or above the criteria (concentration p < 0.01; total sperm count p < 0.001; motility p < 0.0001). Moreover, when these factors exceeded the criteria, the DFI was low for all factors (Fig. 2). When the WHO criteria were not met, the DFI was determined to be moderate or high for all the factors.

The IVF outcome by ICSI in patients who underwent SCD test showed that the low DFI groups had normal fertilization and good blastocyst rates of 82.0% (1053/1284) and 26.0% (334/1284), respectively. The medium DFI group had rates of 80.7% (505/626) and 27.6% (173/626). The high DFI group had rates of 85.9% (152/177) and 17.0% (30/177). There were no significant differences in normal fertilization among the groups. The good-quality blastocyst formation rates were significantly lower in the high DFI group than in the low and medium DFI groups using the ICSI procedure (Table 3). Next, we performed multivariate analysis to adjust for potential confounding bias (Table 4). Multivariate analysis also revealed that a high DFI was associated with only good-quality blastocyst formation (p = 0.0204; adjusted odds ratio, 0.59; 95% confidence interval, 0.38–0.92).

**Table 3:** The relationship between DFI groups and semen analysis

Factors	DFI			p value	
	Low (≤ 15%)	Medium (15–30%)	High (≥ 30%)	Low versus Medium	Low versus High
*Semen analysis*					
Male age (years)	41.2 ± 5.2	43.6 ± 4.5	48.1 ± 4.5	< 0.0001	< 0.0001
Semen volume (mL)	2.8 ± 1.3	3.0 ± 1.3	2.7 ± 1.2	< 0.0001	0.8571
Concentration (10^6^/mL)	67.5 ± 56.1	87.2 ± 100.4	87.4 ± 47.7	0.9828	< 0.0001
Total sperm count	186.4 ± 182.4	209.1 ± 203.6	265.1 ± 205.5	0.8394	< 0.0001
Motility (%)	63.5 ± 13.9	47.3 ± 18.3	30.0 ± 15.4	< 0.0001	< 0.0001
Normal morphology (%)	2.8 ± 1.8	2.5 ± 1.3	2.2 ± 2.1	0.0093	< 0.0001
*ICSI outcome*					
Female age (years)	40.8 ± 3.8	40.9 ± 3.6	42.5 ± 3.4	0.6581	< 0.0001
Normal fertilization (%)	82.0 (1053/1284)	80.7 (505/626)	85.9 (152/177)	0.4789	0.2046
Good quality blastocyst (%)	26.0 (334/1284)	27.6 (173/626)	17.0 (30/177)	0.4508	0.0090
Pregnancy rate (%)	39.4 (52/132)	43.1 (28/65)	36.7 (4/11)	0.6207	1.0000*
Live birth rate (%)	19.7 (26/132)	24.6 (16/65)	36.7 (4/11)	0.4281	0.4249*

Data presented as mean ± standard deviation.

*DFI* DNA fragmentation index; *ICSI* intracytoplasmic sperm injection

*Fisher’s exact test

**Table 4:** Multivariate analysis for ICSI and vitrified-warm blastocyst transfer outcome

		aOR ratio	95% CI	p value
Normal fertilization	Low (reference)	–	–	–
	Medium	0.89	0.69–1.14	0.3552
	High	1.37	0.87–1.67	0.1770
Good blastocyst	Low (reference)	–	–	–
	Medium	1.04	0.84–1.30	0.7073
	High	0.65	0.42–0.99	0.0459
Clinical pregnancy	Low (reference)	–	–	–
	Medium	0.82	0.39–1.69	0.5692
	High	0.60	0.13–2.79	0.5177
Live birth	Low (reference)	–	–	–
	Medium	0.81	0.32–2.03	0.4292
	HIGH	2.42	0.46–12.7	0.2942

*ICSI* intracytoplasmic sperm injection; *CI* confidence interval; *aOdds* adjusted odds ratio

## Discussion

Using SDF evaluation for semen tests enabled the clarification of the correlation between DFI and various factors that are considered to be related to the performance outcome of assisted reproductive technologies. The causes associated with male infertility are still less understood than those associated with women. The semen tests used for evaluation and diagnostics focus primarily on semen volume, concentration, motility, and normal morphology rate. The information obtained from these general semen test findings is considered inadequate for determining sperm fertility potential. In this study, to evaluate the correlation between IVF clinical outcomes and male infertility more accurately, we focused on sperm DFI measurements. The DFI measurement is a method for detecting the integrity of sperm nuclear DNA and parcentatge of damaged sperm. The DFI has been reported to increase with age in men. Le et al. reported in their study that approximately 8% of infertile men have high DFI values (≥ 30%) [14]. A high DFI often results in poor IVF performance. Although there was no significant difference in the normal fertilization rate of ICSI in this study, the good blastocyst development rate was significantly lower in the high DFI group. The embryonic genome is activated at the 4-cell stage, and the influence of parent genes is reflected at the 8-cell stage. Therefore, most researchers believe that sperm DNA damage does not affect oocyte fertilization or embryonic development before the 4-cell stage [15]. Agrawal et al. also reported that sperm DNA damage-associated factors are affected by abnormal sperm lipids, reproductive hormones, and mitochondria [16]. These factors are involved in oxidative stress and apoptosis due to age-dependent decline in male fecundity. This causes an age-related increase in DNA damage [17]. The results of SDF measurements in this study showed that DFI tended to increase with age, as described in previous reports [18–20]. However, DFI is not only affected by age but also by indulgences such as cigarettes [21] and alcohol [22], lifestyle habits such as sleep and exercise, and intake of supplements [23]. We could not evaluate these patient’s lifestyle and preferences in this study. Thus, various aspects of aging, multiple stress factors, and negative factors affecting DFI require further investigations [24]. We also found that the DFI was significantly lower in the group below the criteria than in the group at or above the criteria for concentration, total sperm count, and motility in semen tests conducted in accordance with the WHO criteria [25]. In this study, about 6.0% (8/134) of patient have high sperm DFI levels (≥ 30%). Moreover, the important point of our study is that we revealed that sperm DFI is independent predictor of good-quality blastocyst development using multivariate analysis. It has been reported that although there is no difference in fertilization and embryonic development rates between high and low DFI [26], a high DFI tends to be associated with higher rates of miscarriage, resulting in a low live birth rate per transplantation [27, 28]. These results suggest that DFI evaluation test additional with semen analysis based on WHO criteria was useful for predict ICSI outcome [29]. It may be useful to suspect damage to the sperm nucleus despite patients satisfying or not satisfying the WHO criteria to understand the cause of IVF failure when IVF outcomes are poor.

## Conclusion

In conclusion, SCD testing may lead to the elucidation of potential factors for predicting IVF outcomes. Incorporating the SCD test into the standard semen analysis may result in a more reliable semen diagnostic technique.

In the future, further techniques to evaluate the contribution of sperm DNA to IVF outcomes and to select the best sperm will lead to improved IVF outcomes. Even during the IVF process, certain factors may lead to an increase in DFI, including active oxygen in the semen [30], sperm screening method [31–33], and culturing conditions [34, 35]. Therefore, we will investigate whether optimal sperm selection techniques can be achieved by reducing external stress factors.

## Data Availability

Please contact author for data requests.

## Abbreviations

SDF: Sperm DNA fragmentation
SCD: Sperm chromatin dispersion
DFI: Sperm DNA fragmentation index
WHO: World Health Organization
IVF: In vitro fertilization
